# LASSO-derived nomogram for early identification of pediatric monogenic lupus

**DOI:** 10.1007/s12519-024-00817-y

**Published:** 2024-07-06

**Authors:** Tian-Yu Zhang, Wei Wang, Si-Hao Gao, Zhong-Xun Yu, Wei Wang, Yu Zhou, Chang-Yan Wang, Shan Jian, Lin Wang, Li-Juan Gou, Ji Li, Ming-Sheng Ma, Hong-Mei Song

**Affiliations:** Department of Pediatrics, Peking Union Medical College Hospital, Chinese Academy of Medical Sciences and Peking Union Medical College, Beijing, China

**Keywords:** Children, Lupus, Model, Monogenic, Nomogram, Prediction

## Abstract

**Background:**

Monogenic lupus is defined as systemic lupus erythematosus (SLE)/SLE-like patients with either dominantly or recessively inherited pathogenic variants in a single gene with high penetrance. However, because the clinical phenotype of monogenic SLE is extensive and overlaps with that of classical SLE, it causes a delay in diagnosis and treatment. Currently, there is a lack of early identification models for clinical practitioners to provide early clues for recognition. Our goal was to create a clinical model for the early identification of pediatric monogenic lupus, thereby facilitating early and precise diagnosis and treatment for patients.

**Methods:**

This retrospective cohort study consisted of 41 cases of monogenic lupus treated at the Department of Pediatrics at Peking Union Medical College Hospital from June 2012 to December 2022. The control group consisted of classical SLE patients recruited at a 1:2 ratio. Patients were randomly divided into a training group and a validation group at a 7:3 ratio. A logistic regression model was established based on the least absolute shrinkage and selection operator to generate the coefficient plot. The predictive ability of the model was evaluated using receiver operator characteristic curves and the area under the curve (AUC) index.

**Results:**

A total of 41 cases of monogenic lupus patients and 82 cases of classical SLE patients were included. Among the monogenic lupus cases (with a male-to-female ratio of 1:1.05 and ages of onset ranging from birth to 15 years), a total of 18 gene mutations were identified. The variables included in the coefficient plot were age of onset, recurrent infections, intracranial calcifications, growth and developmental delay, abnormal muscle tone, lymphadenopathy/hepatosplenomegaly, and chilblain-like skin rash. Our model demonstrated satisfactory diagnostic performance through internal validation, with an AUC value of 0.97 (95% confidence interval = 0.92–0.97).

**Conclusions:**

We summarized and analyzed the clinical characteristics of pediatric monogenic lupus and developed a predictive model for early identification by clinicians. Clinicians should exercise high vigilance for monogenic lupus when the score exceeds − 9.032299.

**Graphical abstract:**

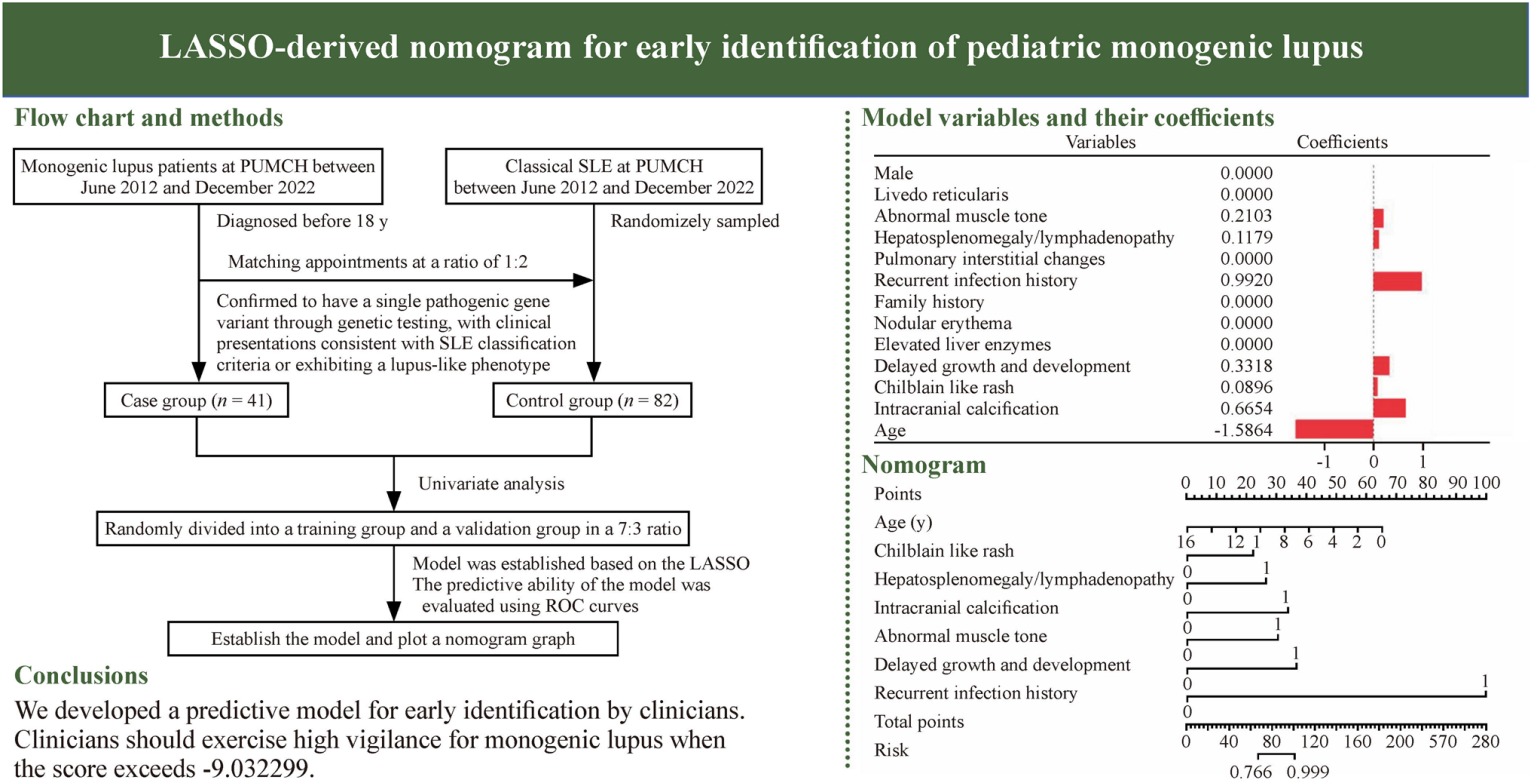

## Introduction

Systemic lupus erythematosus (SLE) is a chronic autoimmune disease, characterized by the presence of autoantibodies and multisystem involvement. Pediatric SLE accounts for about 15% of all SLE patients. The phenotype may be different from adult-onset SLE, with more complex clinical manifestations, rapid progression, and poor prognosis. SLE has a multifactorial pathogenesis; however, the underlying etiology contributions of SLE including genetic, environmental, and immunologic factors have remained elusive.

**Fig. 1:**
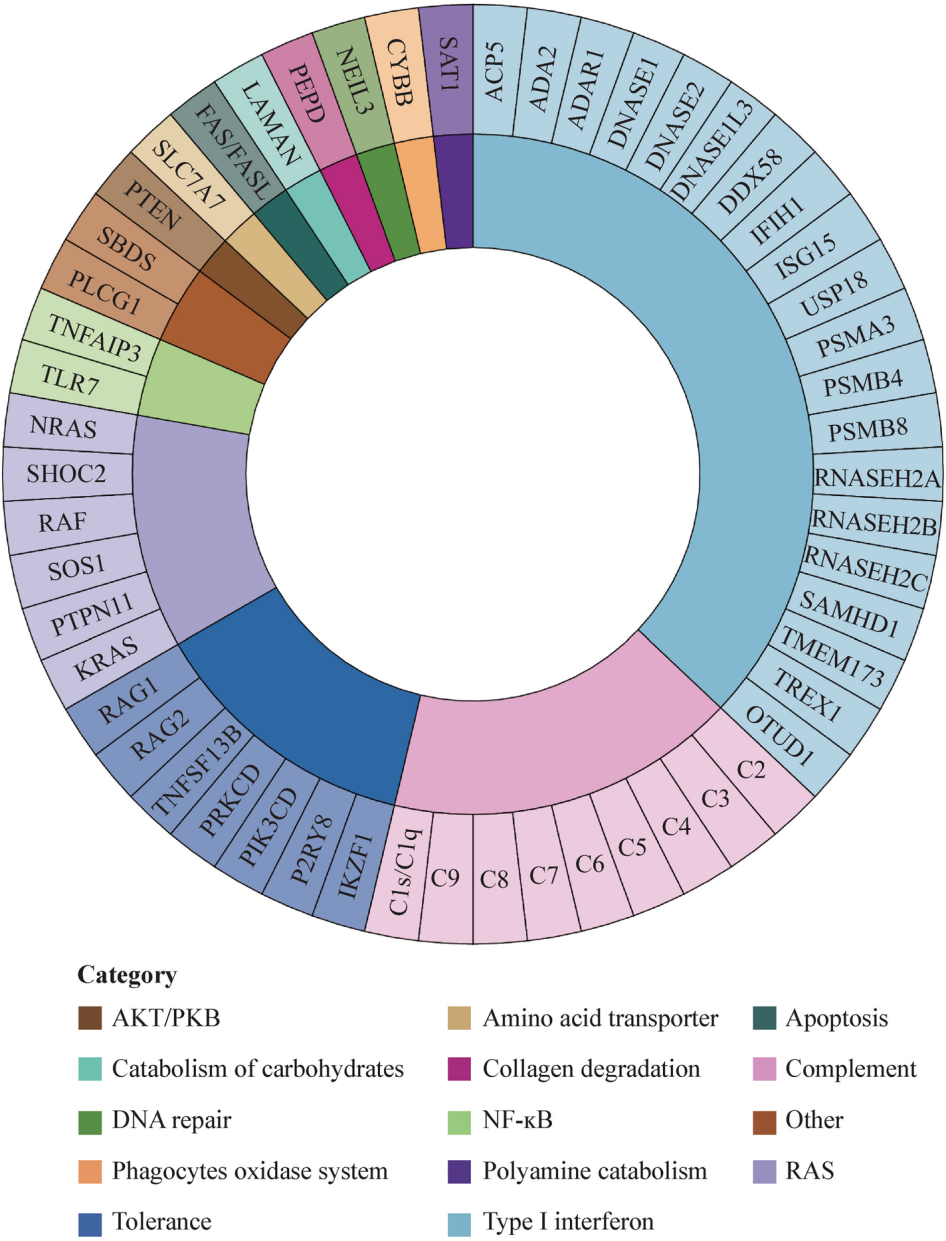
Pathways of genes associated with monogenic lupus. *IKZF1* Ikaros family zinc finger proteins 1, *P2RY8* P2Y receptor family member 8, *PIK3CD* phosphatidylinositol-4,5-bisphosphate 3-kinase, catalytic subunit delta, *RPKCD* protein kinase C delta, *TNFSF13B* tumor necrosis factor superfamily member 13b, *RAG* recombination-activating gene, *KRAS* Kirsten rat sarcoma viral oncogene homolog, *PTPN11* protein tyrosine phosphatase non-receptor type 11, *SOS1* son of sevenless homolog 1, *NRAS* neuroblastoma rat sarcoma viral oncogene homolog, *TLR7* toll-like receptor 7, *TNFAIP3* tumor necrosis factor alpha-induced protein 3, *PLCG1* phospholipase Cgamma 1, *SBDS* Shwachman-Bodian-Diamond syndrome, *PTEN* phosphatase and tensin homolog deleted on chromosome ten, *SLC7A7* solute carrier family 7 member 7, *FAS* Fas cell surface death receptor, *FASL* Fas ligand, *PEPD* peptidase D, *NEIL3* nei endonuclease VIII-like 3, *CYBB* cytochrome b-245, beta polypeptide, *SAT1* spermidine/spermine N1-acetyltransferase 1, *ACP5* tartrate-resistant acid phosphatase, *ADA2* adenosine deaminase 2, *ADAR1* adenosine deaminase RNA specific 1, *DNASE1/2* deoxyribonuclease 1/2, *DNASE1L3* deoxyribonuclease 1 like 3, *DDX58* RNA sensor RIG-I, *IFIH1* interferon induced with helicase C domain 1, *ISG15* interferon-stimulated gene product 15, *USP18* ubiquitin-specific peptidase 18, *PSMA3* proteasome 20S subunit alpha 3, *PSMB4* proteasome 20S subunit beta 4, *RNASEH2A* ribonuclease H2 subunit A, *RNASEH2B* ribonuclease H2 subunit B, *RNASEH2C* ribonuclease H2 subunit C, *SAMHD1* SAM-domain- and HD-domain-containing protein 1, *TMEM173* transmembrane protein 173, *TREX1* three prime repair exonuclease 1, *OTUD1* OTU deubiquitinase 1, *PKB* protein kinase B, *NF-ĸB* nuclear factor-kappa B

Monogenic lupus is defined as SLE patients with either dominantly or recessively inherited pathogenic variants in a single gene with high penetrance [1]. Despite being rare, it has made a significant contribution to revealing the molecular pathogenesis of SLE. Early onset, familial, and syndromic SLE are the clinical features of this disease [2]. Up to 100 susceptibility loci for polygenic SLE, as well as around 50 monogenic causes of SLE or lupus-like phenotype, have been described to date [1–9]. These include pathways in complement deficiencies, type I interferon (IFN) signaling, RAS, and self-tolerance, among others (Fig. 1, as of March 2023) [1, 10]. The vast majority of genetic defects leading to monogenic lupus belong to complement and type I IFN pathways. Considering the complexity of lupus, the recognition of monogenic causes is becoming increasingly important as treatment options are developed based on an understanding of causal molecular pathways. However, it is still difficult for clinicians to identify monogenic lupus at an early stage so effective treatment can be initiated as soon as possible.

The least absolute shrinkage and selection operator (LASSO), as a supervised learning method, is capable of constructing models using labeled data and predicting outcomes for unknown data. A nomogram chart is a convenient and intuitive risk assessment tool that integrates multiple factors. By considering various variables and incorporating the model, it aids healthcare professionals in swiftly and intuitively evaluating a patient’s risk.

We aim to summarize the clinical characteristics of monogenic lupus and develop an early clinical identification model applicable for assessing disease risk in pediatric patients, thus reducing the delay in diagnosing and treating monogenic lupus.

## Methods

### Study design and patients

This study was a retrospective case–control study. Inclusion criteria for the case group were as follows: (1) children under the age of 18 years, treated at Peking Union Medical College Hospital between June 2012 and December 2022, diagnosed with monogenic lupus; (2) confirmed to have a single pathogenic gene variant through genetic testing, with clinical presentations consistent with SLE classification criteria or exhibiting a lupus-like phenotype. Inclusion criteria for the control group were children with classic SLE who were admitted to our hospital within one year before or after the first admission date of the case group, matched in a 1:2 ratio. This study obtained approval from the Ethics Committee of Peking Union Medical College Hospital.

### Definition

The diagnosis criteria for SLE are the 1997 American College of Rheumatology classification criteria or the 2012 Systemic Lupus International Collaborating Clinics classification criteria [11, 12]. A lupus-like phenotype is defined as the presence of involvement of two or more systems in the SLE-affected systems, as well as positivity for one or more autoantibodies, but does not meet the classification criteria for SLE.

### Statistical analysis

Data analysis was conducted using R (version 4.1.1) and GraphPad Prism (version 9.0). Categorical variables were expressed as counts (%). Continuous variables were presented as mean ± standard deviation. A *P* value less than 0.05 was considered statistically significant.

## Results

### Demographic data

We included a total of 41 patients with monogenic lupus and 82 patients with classic lupus. Within the classic SLE group, the average age was 11.65 ± 1.87 years, with nine male patients (male:female ratio of 1:8.1), and six (7.3%) patients had a family history. In the monogenic lupus group, the average age was 3.47 ± 4.18 years, with 20 male patients (male:female ratio of 1:1.05), and nine (22%) patients had a family history. Both groups showed statistically significant differences in terms of age, gender, and family history (*P* < 0.05) (Table 1).

**Table 1:** Demographics of monogenic lupus in 41 patients

Features	Classic SLE (*n* = 82)	Monogenic SLE (*n* = 41)	*P*	OR (95% CI)
Age of diagnosis (y)	11.65 ± 1.87	3.47 ± 4.18	< 0.001	8.18^a^ (7.10–9.25)
Male	9 (11.0)	20 (48.8)	< 0.001	0.13 (0.05–0.33)
Family history	6 (7.3)	9 (22.0)	0.019	3.56 (1.17–10.84)

Data are presented as mean ± standard deviation or count (percentage). *SLE* systemic lupus erythematosus, *CI* confidence interval, *OR* odds ratio. ^a^Median difference

### Gene data

A total of 18 different gene mutations were identified among the 41 pediatric patients, as shown in Table 2. Out of these, 26 cases were associated with type I IFN pathway disorders, including six cases with adenosine deaminase 2 (*ADA2*) gene mutations, five cases with helicase C domain 1 gene mutations, four cases with three prime repair exonuclease 1 gene mutations, three cases with stimulator of interferon gene 1 mutations, two cases with ribonuclease H2 subunit C gene mutations, two cases with tartrate-resistant acid phosphatase gene mutations, two cases with proteasome subunit beta type-8 gene mutations, one case with RNA-editing enzyme adenosine deaminase RNA specific 1 gene mutation, and one case with sterile alpha motif and HD domain-containing protein 1 gene mutation. Additionally, five cases were linked to RAS-associated autoimmune leukoproliferative disorder, comprising two cases with Kirsten rat sarcoma viral oncogene homolog (*KRAS*) gene mutations and three cases with neuroblastoma RAS viral oncogene homolog gene mutations. Furthermore, four cases were related to immune tolerance pathways, with three cases having phosphatidylinositol-4,5-bisphosphate 3-kinase, catalytic subunit delta gene mutations, and one case with an Ikaros family zinc finger proteins 1 gene mutation. There was also one case with C1s deficiency. Other mutated genes included two cases with tumor necrosis factor alpha-induced protein 3 gene mutations, one case with solute carrier family 7 member 7 gene mutation, one case with peptidase D gene mutation, and one case with Shwachman–Bodian–Diamond syndrome (*SBDS*) gene mutation. Some of these gene loci have been previously reported by our team [13–16], while others have been documented in various literature [17–29]. Nine loci from seven genes have not been reported (Table 2). Although two loci of *KRAS* have been reported, they were not related to lupus. In addition, our center reported for the first time that the *SBDS* gene was related to SLE [29].

**Table 2:** Forms of monogenic lupus in 41 patients

Pathway	Gene	Transcript	Inheritance	Mutation	Clinical manifestations
Type I interferon	*TREX1*	NM_016381	AD/AR	c.505C > T, p.R169C c.900delA, p.S301Lfs*31	Nodular erythema, chilblain like rash, intracranial calcification, delayed growth and development
				c.459dupA, p.C154Mfs*3 c.695delA, p.Y232Sfs*3	Intracranial calcification, delayed growth and development, livedo reticularis, abnormal muscle tone, pulmonary hypertension, congenital heart disease
				c.139G > A, p.G47S c.458dupA, p.C154Mfs*3	Chilblain like rash, abnormal muscle tone, intracranial calcification, recurrent infections
				c.45G > T, p.R15S c.139G > A, p.G47S	Livedo reticularis, lymphadenopathy
	*ADAR1*	NM_001111	AR	c.305_306del, p.Q102Rfs*22	Chilblain like rash, intracranial calcification
	*SAMHD1*	NM_015474.4	AD	c.428G > A, p.R143H	Chilblain like rash, hepatomegaly, intracranial calcification, delayed growth and development
	*IFIH1*		AD	c.1016C > A, p.A339D	Chilblain like rash, intracranial calcification, delayed growth and development, pulmonary interstitial involvement
		NM_022168.4		c.2336G > A, p.R779H	Splenomegaly, delayed growth and development, intracranial calcification
				c.2131C > A, p.Q711K	Abnormal muscle tone, intracranial calcification, delayed growth and development
				c.1747A > G, p.I583V	Hepatosplenomegaly, cerebral infarction, intracranial calcification, delayed growth and development
	*RNASEH2C*	NM_032193.4	AR	c.197G > A, p.R66H c.194G > A, p.G65D	Abnormal muscle tone, intracranial calcification, delayed growth and development
				c.401 T > A, p.L134Q^a^ c.194G > A, p.G65D	Abnormal muscle tone, intracranial calcification, delayed growth and development, brain atrophy
				c.562A > C, p.N188H^a^	Intracranial calcification, pulmonary interstitial involvement
	*STING1*	NM_198282.4	AD	c.463G > A, p.V155M	Chilblain like rash, delayed growth and development, pulmonary interstitial involvement
				c.461A > G, p.N154S	Chilblain like rash, pulmonary interstitial involvement, recurrent infections
	*ACP5*	NM_001611.5	AD/AR	c.798dupC, p.S267Lfs*20 c.716G > A, p.G239D	Hepatomegaly, intracranial calcification, brain atrophy, delayed growth and development, recurrent infections, abnormal muscle tone
				c.643G > A, p.G215R c.740 T > C, p.L247P^a^	Flattening of all vertebral bodies in the entire spine, pulmonary interstitial involvement, intracranial calcification, brain atrophy
				c.13G > C, p.G5R Exon7del	Nodular erythema, livedo reticularis, lymphadenopathy
				c.254A > T, p.N85I c.851G > T, p.G284V	Nodular erythema, livedo reticularis, lymphadenopathy, recurrent infections
	*ADA2/CECR1*	NM_001282225.2	AR	c.878A > C, p.H293P c.263A > G, p.Y88C	None
				c.506G > A, p.R169Q c.393delG, p.R131Sfs*52	Livedo reticularis, lymphadenopathy, delayed growth and development
				c.1232A > G, p.Y411C c.983A > T, p.N328I	Nodular erythema, lymphadenopathy, recurrent infections, brain atrophy
				c.1072G > A, p.G358R c.97_c.98insA, p.T33Nfs*2	Splenomegaly
RAS	*NRAS*	NM_002524.5	AD	c.38G > A, p.G13D	Delayed growth and development, hepatosplenomegaly, pulmonary interstitial involvement
				c.37G > T, p.G13C	Recurrent infections, hepatosplenomegaly
	*KRAS*	NM_033360.4	AD	c.437C > T, p.A146V^b^	Hepatomegaly
				c.64C > A, p.Q22K^b^	Hepatosplenomegaly
Tolerance	*PIK3CD*	NM_005026.5	AR	c.3061G > A, p.E1021K	Delayed growth and development, recurrent infections, hepatosplenomegaly
	*IKZF1*	NM_006060	AD	c.488A > C, p.H163P^a^	Delayed growth and development, recurrent infections, hepatosplenomegaly, brain atrophy
NF-KB	*TNFAIP3*	NM_001270508.2	AD	c.133C > T, p.R45X	Recurrent infections, lymphadenopathy
				c.811C > T, p.R271X	Lymphadenopathy, pulmonary interstitial involvement
Proteasome	*PSMB8*	NM_148919.4	AD	c.686G > C, p.R229P^a^ c.421C > T, p.R141X^a^	Chilblain like rash, intracranial calcification
				c.224C > T, p.T75M c.221C > T, p.T74I	Nodular erythema, hepatosplenomegaly, brain atrophy, delayed growth and development
Complement	*C1s*	NM_001734.5	AD/AR	c.849G > A, p.W283X^a^ c.565 + 1G > A (splicing)^a^	Abnormal muscle tone, delayed growth and development
Amino acid transporter	*SLC7A7*	NM_003982.4	AR	c.625 + 1G > A c.182G > T, p.G61V	Delayed growth and development, intracranial calcification, pulmonary interstitial involvement, recurrent infections, hepatosplenomegaly
Collagen degradation	*PEPD*	NM_000285.4	AR	c.15delC, p.G6Dfs*11^a^ c.550C > T, p.R184X	Delayed growth and development, pulmonary interstitial involvement
Other	*SBDS*	NM_016038	AR	c.258 + 2 T > C (splicing)	Recurrent infections

*AD* autosomal dominant, *AR* autosomal recessive, *TREX1* three prime repair exonuclease 1, *ADAR1* adenosine deaminase RNA specific 1, *SAMHD1* SAM-domain- and HD-domain-containing protein 1, *IFIH1* interferon induced with helicase C domain 1, *RNASEH2C* ribonuclease H2 subunit C, *STING1* stimulator of interferon gene 1, *ACP5* tartrate-resistant acid phosphatase, *ADA2* adenosine deaminase 2, *CECR1* cat eye syndrome chromosome region 1, *NRAS* neuroblastoma rat sarcoma viral oncogene homolog, *KRAS* Kirsten rat sarcoma viral oncogene homolog, *PIK3CD* phosphatidylinositol-4,5-bisphosphate 3-kinase, catalytic subunit delta, *IKZF1* Ikaros family zinc finger proteins 1, *NF-ĸB* nuclear factor-kappa B, *TNFAIP3* tumor necrosis factor alpha-induced protein 3, *PSMB8* proteasome 20S subunit beta 8, *SLC7A7* solute carrier family 7 member 7, *PEPD* peptidase D, *SBDS* Shwachman-Bodian-Diamond syndrome. ^a^The loci has not been reported to be associated with lupus; ^b^the gene has not been reported to be associated with lupus

### Clinical characteristics

The primary clinical manifestations of both groups are compared in Table 3. Although skin and mucosal involvement were common in both groups, classic SLE was more likely to present with acute/subacute rashes (67.1%), primarily typical malar rashes (58.5%). In contrast, patients in the monogenic lupus group were more likely to exhibit chronic rashes (39%), particularly chilblain-like rashes (19.5%), nodular erythema (12.2%), and livedo reticularis (19.5%). Hematologic system involvement was more common in classic SLE, mainly manifested as hemolytic anemia (53.7%), whereas monogenic lupus primarily exhibited abnormal liver function (65.9%) in the gastrointestinal system. Neuropsychiatric involvement, on the other hand, was more common in monogenic lupus (46.3%), with abnormal muscle tone being the most common (22%), followed by seizure episodes (14.6%). Monogenic lupus also exhibited more significant respiratory system involvement (53.7%), primarily with pulmonary interstitial involvement (27.8%). Renal involvement was more frequent in the classic lupus group (56.1%). In terms of other clinical presentations, patients with monogenic lupus were more likely to have a history of recurrent infections (31.7%), accompanied by fever (63.4%), lymphadenopathy/hepatosplenomegaly (56.1%), and growth and developmental delay (48.8%). Regarding complications, the classic SLE group was more prone to cytomegalovirus (CMV) infection (70.7%). There were no statistically significant differences in other affected systems.

**Table 3:** Clinical characteristics of monogenic lupus in 41 patients

Features	Classic SLE (*n* = 82)	Monogenic SLE (*n* = 41)	*P*	OR (95% CI)
Mucocutaneous	56 (68.3)	28 (68.3)	1.000	1.00 (0.45–2.24)
Acute/subacute	55 (67.1)	1 (2.4)	< 0.001	0.01 (0.00–0.09)
Facial butterfly rash	48 (58.5)	1 (2.4)	< 0.001	0.02 (0.00–0.14)
Chronic	5 (6.1)	16 (39.0)	< 0.001	9.86 (3.28–29.64)
Nodular erythema	0 (0.0)	5 (12.2)	0.003	1.14 (1.02–1.28)
Chilblain like rash	4 (4.9)	8 (19.5)	0.020	4.73 (1.33–16.79)
Livedo reticularis	1 (1.2)	8 (19.5)	0.001	19.64 (22.36–163.25)
Hematological	62 (75.6)	22 (53.7)	0.014	0.37 (0.17–0.83)
Leukopenia	26 (36.1)	6 (33.3)	0.830	0.89 (0.30–2.64)
Hemolytic anemia	44 (53.7)	6 (14.6)	0.040	0.35 (0.12–0.98)
Thrombocytopenia	21 (25.6)	13 (31.7)	0.476	1.35 (0.59–3.07)
Neuropsychiatric	12 (14.6)	19 (46.3)	< 0.001	5.04 (2.12–11.99)
Abnormal muscle tone	0 (0.0)	9 (22.0)	< 0.001	1.28 (1.09–1.51)
Seizures	2 (2.4)	6 (14.6)	0.016	6.86 (1.32–35.66)
Headache	4 (4.9)	5 (12.2)	0.158	2.71 (0.69–10.69)
Pleuropulmonary	15 (18.3)	12 (53.7)	0.166	1.85 (0.77–4.44)
Pulmonary interstitial involvement	6 (7.3)	9 (22.0)	0.019	3.56 (1.17–10.84)
Gastrointestinal	22 (26.8)	27 (65.9)	< 0.001	5.26 (2.34–11.82)
Abnormal liver function	22 (26.8)	20 (48.8)	0.016	2.60 (1.19–5.69)
Musculoskeletal	43 (52.4)	14 (34.1)	0.055	0.47 (0.22–1.02)
Serositis	15 (18.3)	7 (17.1)	0.868	0.92 (0.34–2.47)
Endocrine	17 (20.7)	12 (29.3)	0.293	1.58 (0.67–3.74)
Thyroid	9 (19.1)	11 (27.5)	0.356	1.60 (0.59–4.37)
Renal	46 (56.1)	9 (22.0)	< 0.001	0.19 (0.08–0.46)
Cardiovascular	13 (17.1)	11 (26.8)	0.210	1.78 (0.71–4.43)
Lymphadenopathy/hepatosplenomegaly	12 (14.6)	23 (56.1)	< 0.001	7.45 (3.13–17.78)
Lymphadenopathy	4 (4.9)	16 (39.0)	< 0.001	12.48 (3.82–40.80)
Hepatomegaly	3 (3.7)	13 (31.7)	< 0.001	12.23 (3.24–46.10)
Splenomegaly	7 (8.5)	12 (29.3)	0.003	4.43 (1.59–12.37)
Delayed growth and development	0 (0.0)	20 (48.8)	< 0.001	1.95 (1.45–2.63)
Recurrent infections	0 (0.0)	13 (31.7)	< 0.001	1.46 (1.19–1.80)
Fever	31 (37.8)	26 (63.4)	0.007	2.85 (1.31–6.20)
CMV infection	58 (70.7)	11 (26.8)	< 0.001	0.15 (0.07–0.35)

Data are presented as count (percentage). *SLE* systemic lupus erythematosus, *CMV* cytomegalovirus, *CI* confidence interval, *OR* odds ratio

While the monogenic lupus group can also exhibit various positive autoantibodies, the classic SLE group is more likely to present with lupus-specific antibodies, such as antinuclear antibodies, anti-double strand-DNA antibodies, anti-Smith antibodies, anti-ribonuclear protein (RNP) antibodies, anti-Sjogren's syndrome antigen A antibodies, anti-ribosomal RNP antibodies, anti-Ro-52 antibodies, anti-complement antibodies, anti-nucleosome antibodies, and lupus anticoagulants. Individuals in the classic SLE group are also more likely to have decreased complement levels, all of which demonstrate significant statistical differences, as shown in Table 4.

**Table 4:** Auxiliary inspection of monogenic lupus in 41 patients

Features	Classic SLE (*n* = 82)	Monogenic SLE (*n* = 41)	*P*	OR (95% CI)
Coomb’s	55 (66.7)	11 (42.3)	0.028	0.37 (0.15–0.91)
ANA	81 (98.8)	25 (62.5)	< 0.001	0.02 (0.00–0.16)
Anti-dsDNA	68 (82.9)	13 (32.5)	< 0.001	0.10 (0.04–0.24)
Anti-Smith	26 (31.7)	5 (13.2)	0.031	0.33 (0.11–0.93)
Anti-RNP	30 (36.6)	6 (15.8)	0.021	0.33 (0.12–0.87)
Anti-SSA	34 (42.0)	6 (15.8)	0.005	0.26 (0.10–0.69)
Anti-SSB	12 (14.6)	2 (5.3)	0.137	0.32 (0.07–1.53)
Anti-rRNP	28 (34.1)	3 (7.9)	0.002	0.17 (0.05–0.59)
Anti-Ro52	15 (18.3)	0 (0.0)	0.003	0.82 (0.74–0.91)
Anti-histone	27 (32.9)	0 (0.0)	< 0.001	0.67 (0.58–0.78)
Anti-nucleosome	29 (35.4)	1 (2.7)	< 0.001	0.05 (0.01–0.39)
Anticardiolipin	20 (24.4)	7 (18.9)	0.510	0.72 (0.28–1.90)
Lupus anticoagulant	22 (26.8)	0 (0.0)	< 0.001	0.73 (0.64–0.83)
β2GP1	16 (19.5)	3 (8.1)	0.116	0.36 (0.10–1.34)
Hypocomplementemia	68 (82.9)	17 (44.7)	< 0.001	0.17 (0.07–0.39)
Elevated ESR	52 (63.4)	25 (61.0)	0.792	0.90 (0.42–1.95)
Intracranial calcification	1 (1.2)	17 (41.5)	< 0.001	57.38 (7.26–453.59)
White matter changes	8 (9.8)	7 (17.1)	0.242	1.90 (0.64–5.68)
Brain atrophy	1 (1.2)	6 (14.6)	0.006	13.89 (1.61–119.66)
Cerebral ischemia	4 (4.9)	4 (9.8)	0.440	2.11 (0.50–8.90)

Data are presented as count (percentage). *SLE* systemic lupus erythematosus, *ANA* antinuclear antibody, *dsDNA* double-stranded DNA, *RNP* ribonuclear protein, *SSA* Sjogren's syndrome antigen A, *rRNP* ribosomal ribonuclear protein, *β2GP1* anti-beta2 glycoprotein 1 antibody, *ESR* erythrocyte sedimentation rate, *CI* confidence interval, *OR* odds ratio

Head imaging findings of the two groups of patients, including head computed tomography, head magnetic resonance imaging/magnetic resonance angiography/magnetic resonance venography, are compared in Table 4. The monogenic lupus group showed a higher proportion of patients with intracranial calcifications and brain atrophy, with significant statistical differences.

### Predictor selection

**Fig. 2:**
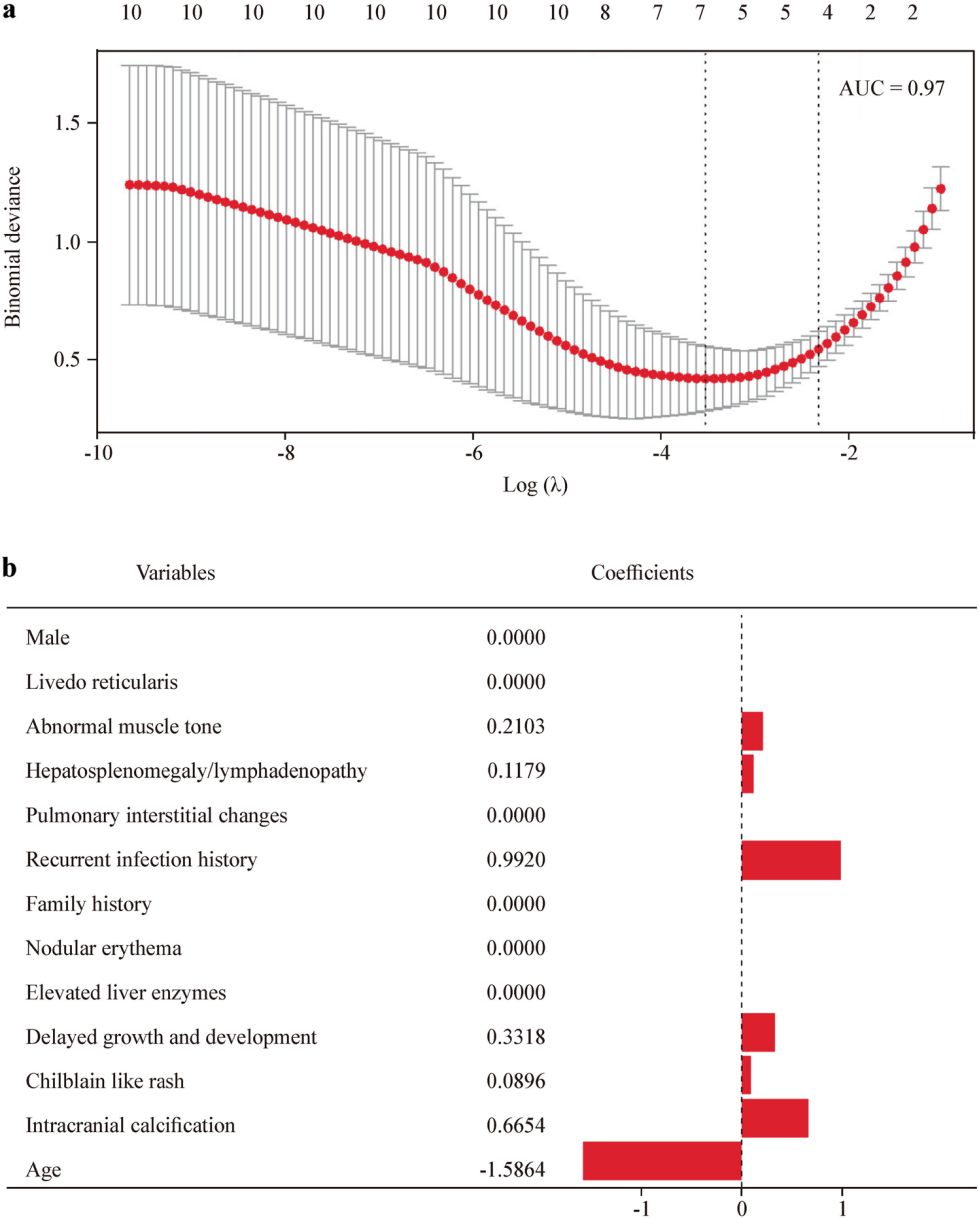
LASSO regression coefficient profiles and model variables with their coefficients. **a** LASSO coefficient profiles of the radiomic featuresl; **b** model variables and their coefficients. *LASSO* least absolute shrinkage and selection operator, *AUC* area under the receiver operating characteristic curve

The variables that showed differences between the two groups in the univariate analysis were included in the LASSO regression analysis. After LASSO regression selection (Fig. 2a), the subsequent seven variables emerged as significant predictive factors for monogenic lupus: age of onset, history of recurrent infections, intracranial calcifications, growth and developmental delay, abnormal muscle tone, lymphadenopathy/hepatosplenomegaly, and chilblain-like skin rash. Notably, the variables in the model are independent. The regression coefficients for these variables were − 1.5864, 0.9920, 0.6654, 0.3318, 0.2103, 0.1179, and 0.0896, respectively (Fig. 2b). When the coefficients are scaled back to the original units of the variables, the resulting linear prediction model for monogenic lupus is as follows: 0.2906977 − 1.4124018 × age of onset + 15.2761768 × recurrent infections + 7.5899250 × intracranial calcifications + 3.5908622 × growth and developmental delay + 3.0491924 × abnormal muscle tone + 1.1751209 × lymphadenopathy/hepatosplenomegaly + 1.2324701 × chilblain-like skin rash.

### Model validation and nomogram construction

**Fig. 3:**
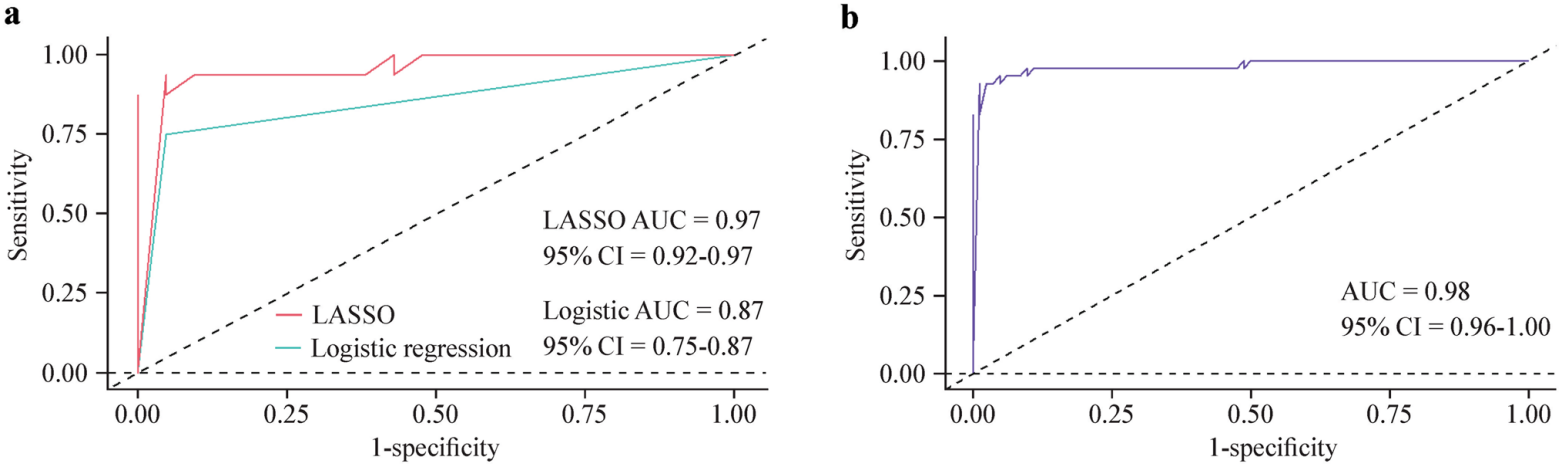
Comparison and risk prediction of monogenic lupus using ROC analysis. **a** The comparison of ROC curves between LASSO regression and logistic regression; **b** ROC curve for risk score prediction of monogenic lupus. *LASSO* least absolute shrinkage and selection operator, *ROC* receiver operating characteristic, *AUC* area under the ROC curve

**Fig. 4:**
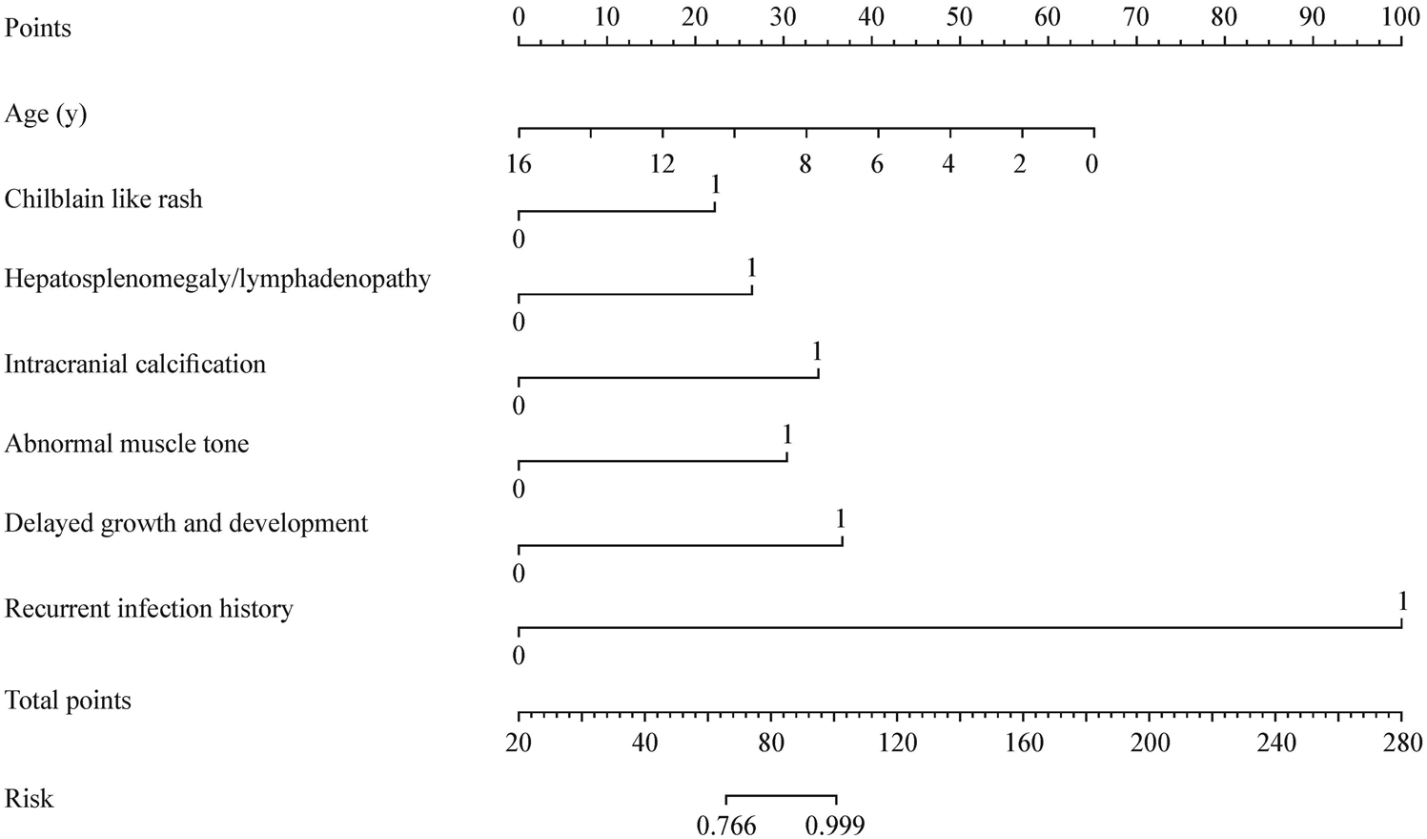
Nomogram for probability of monogenic lupus

The LASSO model achieved an area under the receiver operating characteristic (ROC) curve (AUC) of 0.97 [95% confidence interval (CI) = 0.92–0.97] in the test set (Fig. 3a). We also constructed a logistic regression model and evaluated its performance, resulting in an AUC of 0.87 (95% CI = 0.75–0.87). Comparison between the two models demonstrated the superiority of the LASSO model (Fig. 3a). Furthermore, the LASSO model exhibited an accuracy of 0.86, a precision of 0.81, a recall of 1, and an F1 score of 0.89, indicating excellent predictive performance of this model. We assessed the risk score prediction for monogenic lupus using the ROC curve, which yielded an AUC of 0.98 (95% CI = 0.97–1.00) (Fig. 3b). Utilizing the Youden index, we identified a cutoff score of − 9.032299, suggesting that patients with SLE/lupus-like who have a predictive model score greater than − 9.032299 are at a higher risk of having monogenic lupus, with a risk probability of 0.766 (sensitivity = 92.7%, specificity = 98.8%). We have represented the risk factors in a nomogram (Fig. 4) to facilitate clinicians in intuitively assessing a patient’s risk of developing monogenic lupus.

## Discussion

The understanding of the genetic mechanism of SLE in children has rapidly improved due to the emergence of monogenic lupus. The discovery of new or rare pathogenic gene mutations holds great significance in revealing the molecular pathways involved in the pathogenesis of the disease. Furthermore, such discoveries have the potential to facilitate the provision of individualized treatment. However, because the clinical phenotype of monogenic SLE is extensive and overlaps with that of classical SLE, it causes a delay in diagnosis and treatment. Currently, most reports on monogenic lupus are case studies, and there is a lack of early identification models for clinical practitioners to provide early clues for recognition. Therefore, this study aims to summarize the clinical characteristics of monogenic lupus and establish a clinical early identification model for it. Using a nomogram allows for rapid scoring of patients, indicating the probability of monogenic lupus, enabling clinicians to quickly determine whether genetic testing is necessary and initiate personalized treatment promptly, thereby reducing delays in diagnosis and treatment in clinical practice. Furthermore, for patients who have undergone genetic testing with negative results but score high on the nomogram, it is important not to easily dismiss the possibility of underlying issues. Further analysis, including a comprehensive review of genetic reports, whole genome sequencing or deep sequencing, and somatic cell sequencing, may be warranted to explore potential genetic mutations.

The phenotypic spectrum of monogenic lupus is broad, with different gene mutations leading to pathogenesis through distinct pathways. Currently, reports on its clinical phenotypes are scattered, with most summaries categorized based on the pathogenic pathways [25, 30–35]. Based on our statistical analysis, it has been found that the main clinical manifestations in patients with monogenic lupus were different from those observed in classical SLE.

The aforementioned literature reports that the main clinical manifestations of type I IFN diseases include skin rashes characterized primarily by chilblain-like, livedo reticularis, and nodular erythema. Neurological symptoms typically manifest as abnormal muscle tone and growth and developmental delay. Imaging findings predominantly show intracranial calcifications, white matter changes, and brain atrophy. In addition, other common features include thyroid dysfunction, hepatosplenomegaly, and pulmonary interstitial changes. Our statistical results are generally consistent, except for thyroid dysfunction, which did not show a statistically significant difference between the two groups. Classical lupus is more likely to occur in adolescent girls, whereas monogenic lupus tends to manifest in pre-adolescent children, with roughly equal gender ratios and a higher likelihood of a familial history. Regarding affected systems, monogenic lupus is more prone to chronic skin rashes, especially chilblain-like rashes, nodular erythema, and reticulated purpura. Neurological involvement is more common in monogenic lupus, with abnormal muscle tone being the most common specific manifestation, distinguishing it from classical lupus. However, other common clinical phenotypes seen in type I IFN diseases, such as subclinical thyroid dysfunction, neutropenia, and thrombocytopenia, did not show statistical significance between the two groups upon statistical analysis. Additionally, monogenic lupus more frequently presents with systemic symptoms such as fever and pulmonary interstitial changes. Some clinical features beyond lupus classification criteria are more prominent in monogenic lupus, including lymphadenopathy/hepatosplenomegaly, growth and developmental delay, elevated liver enzymes, and a history of recurrent infections. Furthermore, we observed that classical SLE is more prone to concurrent CMV infection, which is consistent with our previous team’s report [36], potentially providing additional support for the role of CMV in the pathogenesis of classical SLE [37].

A literature review assessed the application of the European Alliance of Associations for Rheumatology 2019 classification criteria in monogenic lupus, suggesting the need for the development of a simplified and effective clinical tool to enable early identification of monogenic lupus [38]. We have constructed the first predictive model for monogenic lupus using the LASSO method: predicted probability = 0.2906977 − 1.4124018 × age of onset + 15.2761768 × recurrent infections + 7.5899250 × intracranial calcifications + 3.5908622 × growth and developmental delay + 3.0491924 × abnormal muscle tone + 1.1751209 × lymphadenopathy/hepatosplenomegaly + 1.2324701 × chilblain-like skin rash. Through the model, it becomes evident that early onset is an indicator of monogenic lupus. Other clinical presentations in the model, such as a history of recurrent infections, intracranial calcifications, growth and developmental delay, abnormal muscle tone, lymphadenopathy/hepatosplenomegaly, and chilblain-like skin rash, should raise a high suspicion of monogenic lupus. Furthermore, we assessed the predictive performance of the risk score for monogenic lupus, which demonstrated good performance (AUC = 0.98, 95% CI = 0.96**–**1.00). Using the Youden index, we identified a cutoff score of − 9.032299, suggesting that patients with SLE who have a predictive model score greater than − 9.032299 are at a higher risk of having monogenic lupus, with a corresponding risk probability of 0.766. In other words, when the risk score is − 9.032299 or higher, there is a 76.6% probability of having monogenic lupus. We have defined a risk probability greater than 0.766 as high risk and created a nomogram chart for clinical use. This tool allows for the convenient calculation of a patient’s risk score, facilitating a rapid assessment of the probability of monogenic lupus. If the risk probability exceeds 0.766, it should raise a strong suspicion of monogenic lupus, prompting the timely completion of genetic testing and additional diagnostic evaluations including the mRNA expression of IFN-stimulated genes and *ADA2* levels, among others.

The main limitations of this study include its single-center retrospective cohort design, with a majority of patients in the cohort having type I IFN diseases. Expanding the dataset and further investigating different disease types is necessary. Additionally, our patient control group did not undergo complete genetic testing. Since this study is retrospective, most of the patients who underwent comprehensive genetic testing clinically had a typical clinical presentations and received genetic testing to establish a definitive diagnosis. We are currently conducting large-scale genetic testing for lupus patients, which can be used for model validation in the next step. Another limitation is that the LASSO method may be overly conservative in selecting sparse models. Although we performed internal validation, the model has not been tested on an external dataset, so its applicability to external data should be confirmed before drawing conclusions.

In conclusion, our study found that age of onset, recurrent infections, intracranial calcifications, growth and developmental delay, abnormal muscle tone, lymphadenopathy/hepatosplenomegaly and chilblain-like skin rash are predictors of monogenic lupus. A model and a nomogram were developed and validated. This nomogram chart can assist clinical physicians in promptly identifying high-risk monogenic lupus patients during their work, thereby reducing delays in the diagnosis and treatment of patients.

## Data Availability

Data are available on reasonable request.
